# Insight into Inhibitor Binding in the Eukaryotic Proteasome: Computations of the 20S CP

**DOI:** 10.3390/ijms19123858

**Published:** 2018-12-03

**Authors:** Milan Hodošček, Nadia Elghobashi-Meinhardt

**Affiliations:** 1 National Institute of Chemistry, Hajdrihova 19, 1001 Ljubljana, Slovenia; milan@cmm.ki.si; 2 Department of Chemistry and Biochemistry, Freie Universität Berlin, 14195 Berlin, Germany

**Keywords:** proteasome, core particle (CP), SyringolinA inhibitor, proteolytic, active site, molecular dynamics (MD), simulations, electrostatic interactions

## Abstract

A combination of molecular dynamics (MD) simulations and computational analyses uncovers structural features that may influence substrate passage and exposure to the active sites within the proteolytic chamber of the 20S proteasome core particle (CP). MD simulations of the CP reveal relaxation dynamics in which the CP slowly contracts over the 54 ns sampling period. MD simulations of the SyringolinA (SylA) inhibitor within the proteolytic $B_{1}$ ring chamber of the CP indicate that favorable van der Waals and electrostatic interactions account for the predominant association of the inhibitor with the walls of the proteolytic chamber. The time scale required for the inhibitor to travel from the center of the proteolytic chamber to the chamber wall is on the order of 4 ns, accompanied by an average energetic stabilization of approximately −20 kcal/mol.

## 1. Introduction

The 26S proteasome complex, found in eukaryotes as well as prokaryotes, is responsible for a range of biological processes, including protein quality control, cell differentiation, antigen processing, signal transduction, cell cycle control, and apoptosis [1]. Together with ubiquitin, the proteasome is responsible for more than 90% of cell protein degradation [1]. The treatment of many life threatening diseases, including certain types of cancer, is based on the selective and efficient inhibition of the proteasome function [1].

**Figure 1:**
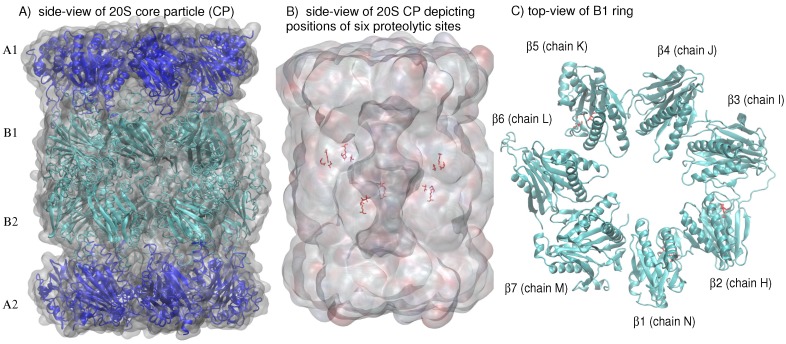
(**A**) A schematic side-view of the 20S proteasome core particle (CP) depicts the four heptameric rings, each consisting of seven subunits, that are stacked on top of each other to form a hollow cylinder. (**B**) A side-view of the 20S CP shows the position of the six proteolytic sites located in the two inner B rings. (**C**) A schematic top-view of the $B_{1}$ ring highlights the ordered arrangement of α-helices and β-sheets in each of the seven subunits; the amino acids (Thr1, Asp17, Lys33) composing the proteolytic sites in $\beta_{1}$ (chain N), $\beta_{2}$ (chain H), and $\beta_{5}$ (chain K) are colored in red (stick representation).

In eukaryotes, the 26S proteasome (drawing its name from its Svedberg (S) sedimentation coefficient as determined by density-gradient centrifugation analysis [2]) is composed of the 20S proteasome core particle (CP) and two 19S capping complexes which have a regulatory function [3]. About 150 Å in height and 110 Å in diameter, the 670-kilodalton 20S CP, or multicatalytic protease complex, consists of four heptameric rings, each containing seven subunits, that are stacked on top of each other to form a hollow cylinder [3]. The A rings, comprised of the α subunits, form the outer cylinder rings while the B-rings, comprised of the β subunits, form the inner rings (Figure 1A). The α and β subunits of the eukaryotic proteasome differ in sequence, with the structural hallmark of the α subunits being the NH${}_{2}$-terminal extension of about 35 amino acid residues [3]. The interfaces between the A and B rings are the gates to the antechambers, where peptide substrates travel to undergo proteolysis [4]. Only three of the seven β subunits contain N-terminal proteolytic active centers, $\beta_{1}$, $\beta_{2}$, and $\beta_{5}$ (depicted schematically for the $B_{1}$ ring in Figure 1B,C), with caspase-like, trypsin-like, and chymotrypsin-like proteolytic activities, respectively [5].

**Figure 2:**
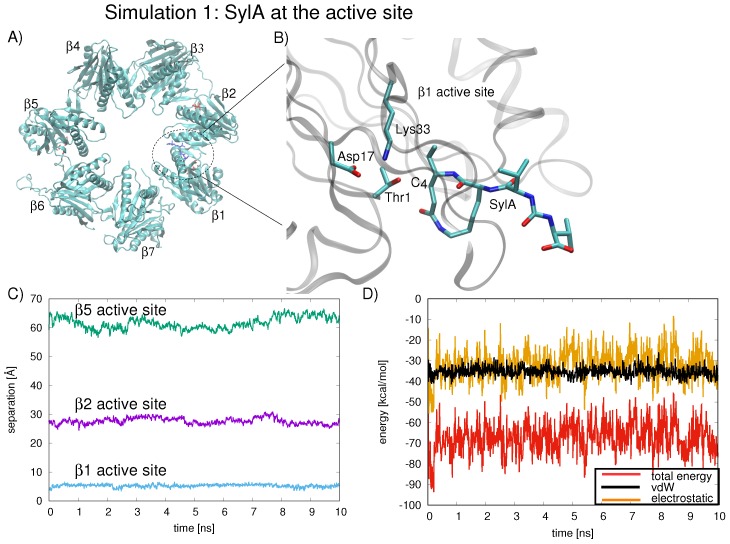
(**A**) Snapshot of the $B_{1}$ ring shown from above, highlighting active site residues (red stick representation) and SylA inhibitor (blue stick representation) at t = 0.0 ns (circled in dotted line), as co-crystallized in the proteasome near the active site of the $\beta_{1}$ subunit (PDB ID 2ZCY [6]). (**B**) Up-close view of the region within the dotted line showing SylA inhibitor at t = 0.0 ns, as co-crystallized in the proteasome near the active site of the $\beta_{1}$ subunit (PDB ID 2ZCY [6]), is depicted, with residues (Thr1, Asp17, and Lys33) involved in binding the SylA inhibitor labeled. For clarity, SylA and active site nitrogen atoms and oxygen atoms are colored blue and red, respectively; the remainder of the protein is depicted as gray ribbon. (**C**) The position of the SylA inhibitor during the MD is quantified by the separation between C${}_{4}$ and Thr1-O${}^{\gamma }$ of each active site, $\beta_{1}$ (blue line), $\beta_{2}$ (purple line), and $\beta_{5}$ (green line). (**D**) The total interaction energy between SylA and all seven $B_{1}$ subunits energy (red line) can be decomposed into van der Waal’s interactions (black line) and electrostatic interactions (orange line). Favorable electrostatic (orange line) and vdW (black line) energies are observed during the duration of the 10 ns simulation, in which the inhibitor is localized at the $\beta_{1}$ active site.

The proteolytic and autocatalytic activities of the 20S proteasome have been well characterized [5]. Using X-ray crystallography and biochemical assays, Huber et al. analyzed the mechanism of $\beta_{1}$, $\beta_{2}$, and $\beta_{5}$ activation. Two sets of catalytic triads were identified: (1) after assembly of the 20S complex, the N-terminal Thr1 of the active site is deprotonated by Lys33 working with Asp17, and (2) Asp166OH, acting with Ser129OH as a proton shuttle, protonates the amine group of Thr1 [5]. The strict conservation of Thr in proteasomes is attributed to the hydrophobic interactions that anchor the C${}^{\gamma }$ of Thr1 with Ala46 (C${}^{\beta }$), Lys33 (carbon side chain), and Thr3 (C${}^{\gamma }$) [5]. The positively charged Thr1NH${}_{3}^{+}$ terminus hydrogen bonds to the amide nitrogen of the incoming peptide and stabilizes it (active site residues depicted in Figure 2B), preparing the substrate for endoproteolytic cleavage by Thr1O${}^{\gamma }$ [5].

**Figure 3:**
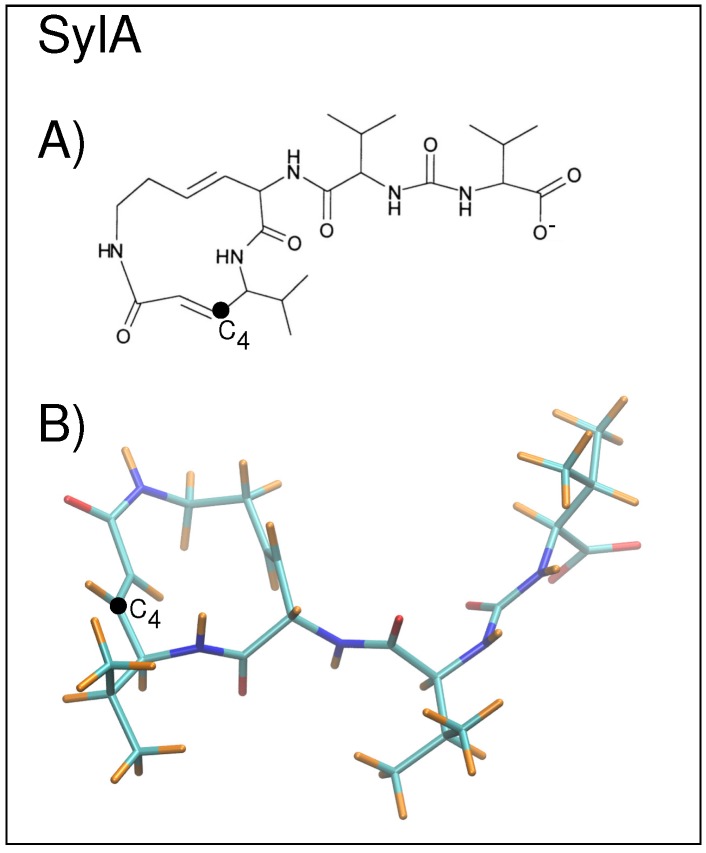
(**A**) A stick diagram of the SylA inhibitor is shown with C${}_{4}$ labeled; (**B**) the non-planar structure of SylA is shown with carbons in cyan, oxygens in red, nitrogens in blue, and hydrogens in orange; C${}_{4}$ is labeled.

Several classes of inhibitors exist, including peptide aldehydes, peptide boronates, peptide vinyl sulfones, peptide epoxyketones, and lactacystin and derivatives thereof [1]. Recently, a plant pathogen virulence factor, syringolin A (SylA) was shown to irreversibly inhibit all three catalytic activities of eukaryotic proteasomes [6] (Figure 3). The inhibition mechanism proceeds via a covalent binding of the hydroxy group of the active site amino (N)-terminal Thr1 to the SylA double bond located at the C${}_{4}$ position [6,7]. Co-crystallization of SylA with the 20S proteasome (2.90 Å resolution) showed binding at all six active sites of the proteasome (pdb structure 2ZCY) [6]. Structural studies have also been carried out on SylB, which differs from SylA by the substitution of the SylA 3,4-dehydrolysine residue with a lysine moiety, to compare selectivity and potency of proteasome inhibition [8]. SylB was found to bind only to subunits $\beta_{2}$ and $\beta_{5}$, whereas SylA binds to all three catalytic subunits [8].

Detailed reaction pathways and free energy profiles for the inhibition reaction of the proteasome (catalytic subunit $\beta_{5}$) have been computed for SylA and another peptide inhibitor, epoxomicin using model systems consisting of the $\beta_{5}$ and $\beta_{6}$ subunits [7,9]. Nonetheless, the mechanism that governs the trafficking of substrates from the outer A ring annulus to the inner proteolytic chamber of the B ring is still not well understood. What forces drive the incoming peptidic substrate to the cavity walls and ultimately to the proteolytic active sites? Experimental and theoretical studies have tried to unravel the complex machinery of the giant proteasome. Magnetization exchange NMR spectroscopy has been used to study the kinetics of the gating mechanism [10]. There, a single A ring (180 kDalton) was used as a model to study the A ring gate transition. By comparing rates of gate exchange for viscogens of different sizes, the authors demonstrated that the gating event proceeds through very small step sizes, involving small protein segments, and that water plays a critical role during this process [10]. The gating event is thought to take place over a series of small steps that involve an effective hydrodynamic radius (EHR) that is <3.5 Å. [10]. Using Kramers’ theory in the strong friction limit [11], the authors determined that the rate constant for the gating event ranges between 600 ms and 40 ms, in the high viscosity limit [10]. One notable finding was that the internal friction forces (originating from the protein) are on the order of or smaller than the viscosity of water at 45 ${}^{\circ }$C. The authors conclude that the collisions with water molecules are essential for the gating process.

Using NMR spectroscopy, Ruschak et al. investigated the interaction of the 20S CP with three small protein substrates [12]. Using a model system composed of two stacked A rings, the authors conclude that the proteins must unfold to enter the 13 Å diameter of the ring annulus; once inside the antechamber (cavity of the A rings), the substrates remain unfolded, strongly interacting with the proteasome’s cavity walls [12]. The protein conformations are an ensemble of interconverting, unstructured states which is best suited for efficient processing by the proteolytic sites [12].

A recent theoretical investigation used molecular dynamics to study the role of the N-termini tails of the α subunits on the gating mechanism [13]. There, long MD (100 μs) simulations were carried out together with a nine-residue polypeptide (Arg-Pro-Pro-Gly-Phe-Ser-Ala-Phe-Lys) whose sequence is often used to analyze substrate hydrolysis [13]. The author could show a moderate attraction of the substrate to the inner wall of the antechamber, in agreement with observations of the NMR experiment [12]. These interactions could also influence the substrate’s behavior in the proteolytic chamber ($B_{1}$ and $B_{2}$) [13]. The author also concluded that the dynamics of the N-termini tails were entropically favorable for the translocation of the substrate.

Here, we aim to characterize further the structural features of the 20S proteasome CP that may be responsible for guiding peptide substrates to an active site in the proteolytic chamber of the B rings. On the nanosecond timescale that was considered here, protein side-chain dynamics can be studied [4]. Characterization of the proteasome and its active sites is essential for understanding not only the machinery of the 20S CP but also for the design of effective proteasome inhibitors. We used a combination of molecular modeling, electrostatic energy calculations, and molecular dynamics (MD) to investigate the proteasome with and without the presence of an inhibitor substrate. To study inhibitor dynamics, the $B_{1}$ ring was simulated with the SylA inhibitor in the absence of the remaining three CP rings. This “dissection” approach, i.e., studying an isolated ring of the CP, has been verified experimentally and is considered to be a valid approximation of the ring dynamics in the assembled CP [4,10]. In the next section, we will discuss in detail the construction of the models and the computational methods that we used for each of these modes of investigation.

## 2. Results and Discussion

**Figure 4:**
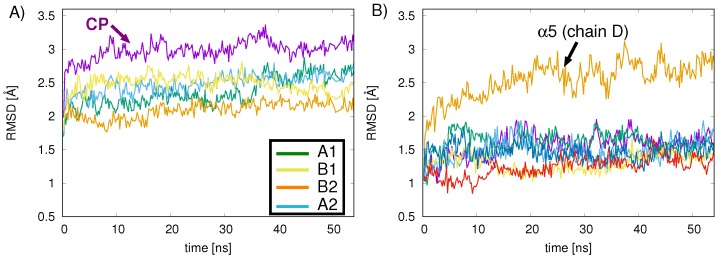
(**A**) protein The backbone RMSD (Å) relative to the crystal structure is shown over the 54 ns simulation time for the apo 20S CP (purple) and for the four ring systems $A_{1}$ (green), $B_{1}$ (yellow), $B_{2}$ (orange), and $A_{2}$ (blue) and (**B**) for the $A_{1}$ subunits: $\alpha_{1}$ (chain G) red, $\alpha_{2}$ (chain A) purple, $\alpha_{3}$ (chain B) green, $\alpha_{4}$ (chain C) light blue, $\alpha_{5}$ (chain D) orange, $\alpha_{6}$ (chain E) yellow, $\alpha_{7}$ (chain F) dark blue. Subunit $\alpha_{5}$ (chain D) (orange), indicated with the arrow, shows the largest RMS deviations over the simulation time period, arising from less structured loops.

54 ns MD simulations of the 20S CP proteasome were carried out to investigate the stability and relaxation dynamics of the CP. The relaxation shows moderate (∼3 Å) root-mean-square deviations (RMSDs) for protein backbone atoms relative to the crystal structure over the 54 ns trajectory (Figure 4A). To analyze the origin of these structural changes, we compared the RMSD deviations relative to the crystal structure for the individual subunits, for the four rings, and for the entire proteasome (Figure 4A). We observe smaller RMSD values (∼2–2.5 Å) for the individual CP rings, as compared to the entire CP structure (purple line in Figure 4A), and for most of the individual ring subunits (α subunits are shown in Figure 4B as an example) RMSD values around 1.5 Å. For subunits $\alpha_{5}$ and $\alpha_{4}^{\prime }$ (chains D and R, respectively), the RMSD difference from the backbone atoms of the crystal structure shows the largest (2.5–2.9 Å) deviations. The source of these RMSD deviations was examined by analyzing the secondary structure of the $\alpha_{5}$ subunit. Less structured loops of the subunit, corresponding to residues 1–12, 46–54, and 115–127, are responsible for raising the subunit’s overall average RMSD (2.5 Å); the remaining residues of the $\alpha_{5}$ subunit are more structured and exhibit an overall average RMSD of 1.4 Å.

**Figure 5:**
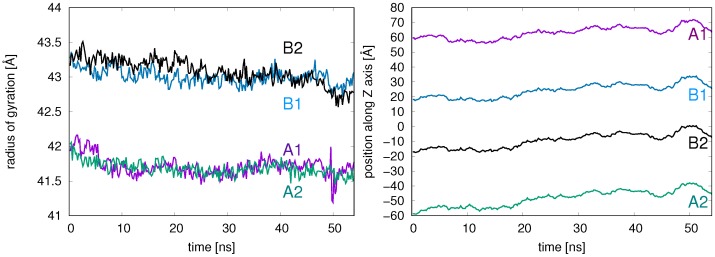
(**A**) The radius of gyration (Å) and (**B**) the position along the *Z*-axis of each ring’s C.O.M. is shown for each of the four ring systems $A_{1}$ (purple), $B_{1}$ (blue), $B_{2}$ (black), and $A_{2}$ (green) over the 54 ns simulation time.

From these differences in RMSD values, one may conclude that the overall structure of the ring subunits is preserved over the course of the MD simulations while positioning of the subunits of the 20S CP undergoes a conformational change relative to the starting structure. These changes may be viewed as relaxation dynamics, in which the CP slowly contracts, but longer simulation times would be necessary to assess this dynamical behavior definitively. The source of this relaxation dynamics was analyzed by calculating the radius of gyration and the relative position (along the *Z* axis) of the center of mass (C.O.M.) for each of the four rings (Figure 5A,B). The radius of gyration of the outer rings, $A_{1}$ and $A_{2}$, is smaller than that of the inner rings due to the presence of the NH${}_{2}$-terminal tails that extend toward the ring center. The A rings exhibit a slight decrease (0.25–0.50 Å) in radius of gyration over the sampling period, and the two inner rings, $B_{1}$ and $B_{2}$, with a larger radius of gyration, also show changes in ring compactness. Interestingly, at t = 54 ns, the C.O.M. of $A_{1}$, Z = +63 Å, is close to its initial position of Z = +60 Å (purple line in Figure 5B) while the $A_{2}$ C.O.M. position (green line in Figure 5B) shifts nearly 10 Å upward toward $B_{1}$, from Z = −60 Å to Z = −50 Å. Similarly, $B_{2}$ demonstrates a shift of nearly 10 Å upward toward $B_{1}$. The overall result of these ring shifts is a slight compression of the proteasome over the 54 ns relaxation period. Again, longer simulation times are required in order to assess this dynamical behavior. The compression could be a relaxation process or a snapshot of a dynamical “breathing” in which the CP compresses and expands over long times.

**Figure 6:**
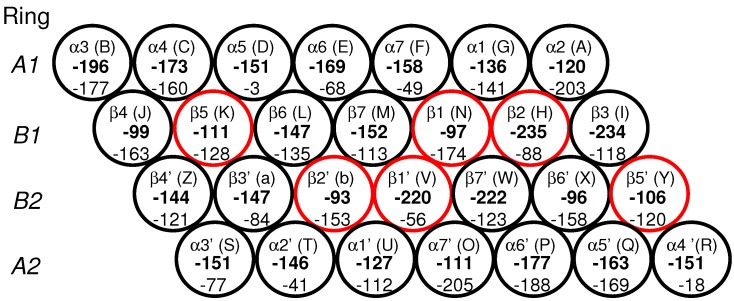
The diagram shows the arrangement of the 28 subunits within the four heptameric rings, $A_{1}$, $B_{1}$, $B_{2}$, and $A_{2}$, of the CP of the 20S proteasome. The subunits containing proteolytic active sites are outlined in red. The chain labels, according to PDB 5CZ4 [5], are listed in parentheses for clarity. Electrostatic energies (kcal/mol) (bold numbers) and solvation binding energies (kcal/mol) are shown for each subunit.

The stability of the 20S CP was next analyzed by computing the electrostatic energies of all subunits in the four heptameric rings. Figure 6 reports the calculated electrostatic energy for each subunit. In the $A_{1}$ ring, the energies vary between −120 kcal/mol and −196 kcal/mol. The largest deviation of these energies occurs between subunits $\alpha_{2}$ (chain A) and $\alpha_{3}$ (chain B), for which the $\Delta E=E\left(\alpha_{2}\right)-E\left(\alpha_{3}\right)=$ 76 kcal/mol. This dip in potential energy is located above the two subunits with the lowest electrostatic energy in the $B_{1}$, subunits $\beta_{2}$ (chain H) and $\beta_{3}$ (chain I), −235 and −234 kcal/mol, respectively. Both subunits (chains H and I) are flanked by subunits whose electrostatic energies are the highest of all seven subunits in the $B_{1}$ (chain J is −99 kcal/mol and chain N is −97 kcal/mol). In other words, the potential energy climb from the active site subunits to the left or to the right is approximately 135 kcal/mol (Figure 6). The third active site subunit, $\beta_{5}$ (chain K), has a moderate energy of −111 kcal/mol. A similar pattern is observed in the $B_{2}$ ring. Subunit $\beta_{7}^{\prime }$ (chain W)(−220 kcal/mol) is flanked on one side by $\beta_{6}^{\prime }$ (chain X) with a higher electrostatic energy (−96 kcal/mol) and on the other side by $\beta_{1}^{\prime }$ (chain V) with a similar electrostatic potential (−220 kcal/mol). The $\beta_{1}^{\prime }$ (chain V) subunit, containing an active site, is flanked on the other side by a subunit with a much higher electrostatic energy (−93 kcal/mol).

The pattern of electrostatic energy distribution in the outer $A_{2}$ ring is quite similar to that of the $A_{1}$ ring. The largest deviation between neighboring subunits occurs between $\alpha_{6}^{\prime }$ (chain P) and $\alpha_{7}^{\prime }$ (chain O), for which the $\Delta E=E\left(\alpha_{6}^{\prime }\right)-E\left(\alpha_{7}^{\prime }\right)$ is 66 kcal/mol. This dip in electrostatic energy is located directly below the subunit $\beta_{7}^{\prime }$ (chain W) with the lowest electrostatic energy in the $B_{2}$ ring.

To check whether electrostatic energy distributions may be related to the binding strength of the subunits within the ring, we calculated the binding solvation energy (Figure 6). The weakest binding energies of all 28 subunits were calculated for $\alpha_{5}$ (chain D) (−3 kcal/mol), and $\alpha_{4^{\prime }}$ (chain R) (−18 kcal/mol) (see Section 3.4). These two subunits, $\alpha_{5}$ and $\alpha_{4^{\prime }}$, are both positioned above/below the $\beta_{5}$ (chain K) and $\beta_{5^{\prime }}$ (chain Q) subunits containing an active site (see subunit arrangement in Figure 6). Interestingly, the $\alpha_{5}$ and $\alpha_{4^{\prime }}$ subunits in the original PDB were the only subunits missing internal residues. The lack of structural information for these two subunits could reflect the weak binding strength or a region of relatively high mobility, perhaps due to gateways of substrate entry/exist.

In the A rings, the strongest binding energies were calculated for $\alpha_{2}$ (chain A) and $\alpha_{7^{\prime }}$ (−203 kcal/mol and −205 kcal/mol, respectively). The strongest binding energies in the B rings are $\beta_{4}$ (chain J) and $\beta_{6^{\prime }}$ (chain X) (−163 kcal/mol and −158 kcal/mol). Both subunits are flanked on the side and above or below by a subunit containing an active site (see Figure 6). One can speculate that strongly bound subunits may be responsible for maintaining the local geometry of the subunits near active site subunits. Of the B rings, the two subunits with the highest average B-factor (Table A1 in Appendix A), $\beta_{2}$ (chain H) and $\beta_{1^{\prime }}$ (chain V) (both active site-containing subunits), have the weakest binding energy (−88 kcal/mol and −56 kcal/mol, respectively), possibly related to the need for these subunits to be flexible enough to accommodate substrates. Variations in electrostatic energies and binding strengths thus reflect differences in A and B ring architectures that may lead to dynamical conformational changes important for substrate processing.

Next, we examined the dynamics of the SylA inhibitor inside the proteolytic chamber by carrying out MD simulations of the SylA with the smaller model system, the proteolytic ring $B_{1}$. In the first simulation, at t = 0 ns, the inhibitor is localized at the $\beta_{1}$ active site. In the three further independent simulations, at t = 0 ns, the inhibitor is located in the ring center. We quantify the average position of SylA with respect to the three active sites throughout the 10 ns simulation as the separation of SylA C${}_{4}$ (involved in covalent bonding with Thr1-O${}^{\gamma }$) from the Thr1-O${}^{\gamma }$ of each binding site, $\beta_{1}$, $\beta_{2}$, and $\beta_{5}$.

In the first simulation (simulation 1, Figure 2A), at t = 0 ns, the inhibitor is localized in the region of the $\beta_{1}$ active site (see Figure 2B for scheme of SylA located at $\beta_{1}$ active site). The inhibitor remains anchored at this position for the duration of the 10 ns simulation (Figure 2C, separation of SylA C${}_{4}$ from the Thr1-O${}^{\gamma }$ of $\beta_{1}$, $\beta_{2}$, and $\beta_{5}$ shown in blue, green, and purple lines, respectively). The interaction energy between SylA and all seven $B_{1}$ subunits was calculated for the 10 ns simulation; the average interaction energy is −67.3±7.5 kcal/mol (Figure 2D, red line). The total interaction energy can be broken down into van der Waal’s interactions (Figure 2D, black line) and electrostatic interactions (Figure 2D, orange line), both of which are favorable throughout the 10 ns sampling period, explaining the stability of the inhibitor’s position at the active site of $\beta_{1}$.

**Figure 7:**
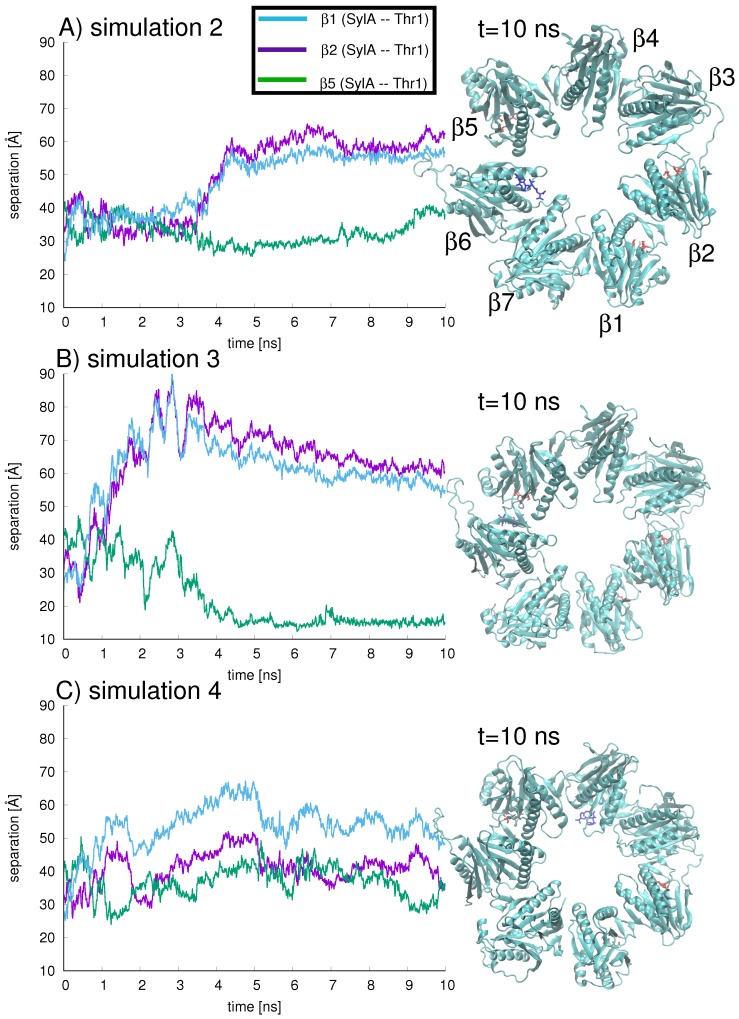
Three independent simulations, labeled simulation 2, 3, and 4 (shown in figures A–C, respectively), were carried out with the $B_{1}$+SylA complex over 10 ns. In each simulation, the SylA position with respect to the three binding sites is quantified by the separation of SylA C${}_{4}$ from the Thr1-O${}^{\gamma }$ of $\beta_{1}$ (blue), $\beta_{2}$ (purple), and $\beta_{5}$ (green). Snapshots of the SylA inhibitor (dark blue) inside the $B_{1}$ ring (active sites depicted in red) are shown for each simulation at t = 10 ns.

Next, to model the scenario in which the inhibitor may migrate from the ring center towards the proteasome wall, three independent trajectories (simulations 2–4) were started for the $B_{1}$+SylA complex in which the initial position of the SylA inhibitor was the center of the ring (Figure 7A–C). Again, the inhibitor position with respect to the three binding sites is quantified by the separation of the SylA C${}_{4}$ atom from the Thr1-O${}^{\gamma }$ atom of each binding site, $\beta_{1}$ (blue), $\beta_{2}$ (purple), and $\beta_{5}$ (green), shown in Figure 7.

All three simulations demonstrate that, starting from the ring center at t = 0 ns, the inhibitor migrates to a position nearer to the proteasome wall. In other words, SylA is never localized in the ring center, so the probability of being near an active site is increased. However, over the 10 ns sampling time, the inhibitor is never located directly at a binding site, so an affinity with the set of catalytic amino acids cannot be claimed. The simulation showing the closest association between SylA and an active site is simulation 3, for which after 4 ns SylA is located closest to the proteolytic site in subunit $\beta_{5}$ (green line in Figure 7B) and farthest from proteolytic subunits $\beta_{1}$ and $\beta_{2}$ (Figure 7B, blue and purple lines, respectively). This position is maintained throughout the 10 ns simulation (snapshot in right-hand panel of Figure 7B).

In simulation 2, around 4 ns the inhibitor (shown in dark blue stick representation in Figure 7A) has traveled to subunit $\beta_{6}$, adjacent to the proteolytic site at $\beta_{5}$, with a separation ca. 40 Å between SylA C${}_{4}$ and Thr1-O${}^{\gamma }$ of $\beta_{5}$ (Figure 7A, green line). The separation from the proteolytic sites in subunits $\beta_{1}$ (Figure 7A, blue line) and $\beta_{2}$ (Figure 7A, purple line) is around 60 Å. At 10 ns (snapshot in right-hand panel of Figure 7A), the inhibitor is still located close to subunit $\beta_{6}$.

**Figure 8:**
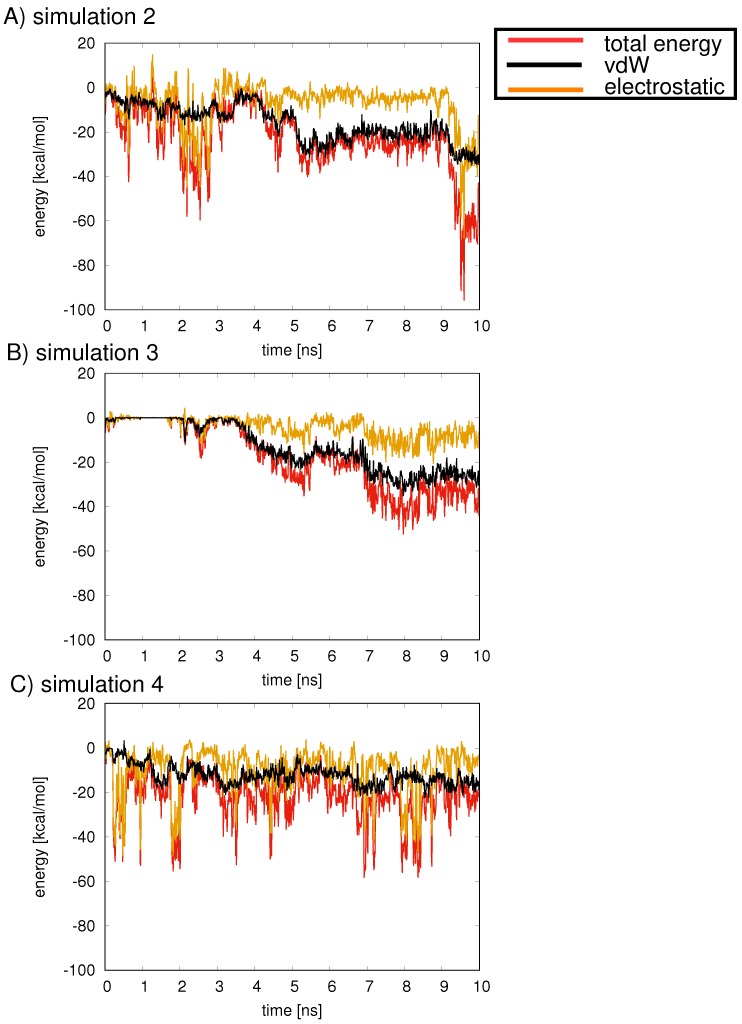
For each of the three independent simulations discussed in Figure 7, labeled simulation 2, 3, and 4 (shown in figures A, B, and C, respectively), the interaction energy [kcal/mol] (red) of SylA with all seven $B_{1}$ subunits was calculated over the length of the trajectory. A breakdown of the energies into van der Waals (black) and electrostatic (orange) components is shown.

In simulation 4 (Figure 7C), the inhibitor visits regions near $\beta_{4}$, nearly equidistant (ca. 36 Å at t = 10 ns) from $\beta_{2}$ and $\beta_{5}$, with some fluctuations in position (Figure 7C, purple and green lines, respectively); at t = 10 ns, SylA is farthest (ca. 48 Å) from $\beta_{1}$ (Figure 7C, blue line) and located at the interface between $\beta_{3}$ and $\beta_{4}$. This region is characterized by a large gradient in electrostatic energy (see Figure 6) which may attract the inhibitor. To check the forces driving the inhibitor’s dynamical position, the interaction energy between the SylA inhibitor and all seven $B_{1}$ subunits was calculated for each of the three trajectories (shown in Figure 8A–C).

For all simulations, an energetic stabilization is observed as the inhibitor moves from the center of the ring to the ring walls. In simulation 2, SylA is in close proximity to the protein chains of $\beta_{6}$, leading to the favorable electrostatic and van der Waals energies (Figure 8A, orange and black lines, respectively). Simulation 2 briefly exhibits the lowest total energy, close to −100 kcal/mol (Figure 8A, red line), near 9.6 ns, followed by simulation 4 with frequent low energy (ca. −50 kcal/mol) configurations (Figure 8C, red line). Simulation 3 shows one low energy configuration (−50 kcal/mol around 8 ns) (Figure 8B, red line). In general, the electrostatic interactions (Figure 8A–C, orange lines) comprise the dominant energetic contribution to the stabilizing energy between inhibitor and ring subunits, while the van der Waals energies (Figure 8A–C, black lines) provide relatively moderate energetic stabilization.

One can compare the energetics of simulations 2–4 over the 10 ns sampling period to those observed in simulation 1, in which SylA is located at the active site of $\beta_{1}$ (Figure 2D). In simulation 2, the average interaction energy of SylA with the protein subunits is only −23.0 ± 14.8 kcal/mol (compare with −67.3 ± 7.5 kcal/mol found in simulation 1). For simulations 3 and 4, average SylA+$B_{1}$ interaction energies of −17.9 ± 14.7 kcal/mol and −22.1 ± 10.9 kcal/mol, respectively, are calculated over the t = 10 ns sampling period. Thus, an energetic stabilization is observed as the inhibitor migrates from the ring center to the walls of the proteolytic chamber. Nonetheless, the chemical environment that is realized when SylA is positioned at an active site, as in simulation 1, is not observed in simulations 2–4, reflected in the less favorable electrostatic and van der Waals interactions. Longer sampling may be required to observe the migration of the inhibitor to an active site position. Also, the electrostatic environment of the model system of $B_{1}$ may not sufficiently reproduce that of the double B ring system, which in turn influences the dynamical behavior of the inhibitor inside the proteolytic chamber.

## 3. Methods

### 3.1. Construction of Models

The 20S proteasome CP was modeled using the PDB 5CZ4 structure (2.30 Å resolution) [5]. The structure consists of 28 subunits; $A_{1}$ (1-7), $B_{1}$ (1-7), $A_{2}$ (1-7), and $B_{2}$ (1-7). Here, the two outer rings will be given the notation $A_{1}$ and $A_{2}$; the inner two rings will be denoted as $B_{1}$ and $B_{2}$. The subunits of the outer rings will accordingly be denoted with lowercase α while the subunits of the inner rings, $B_{1}$ and $B_{2}$, are denoted with β. The 28 subunits have corresponding chain labels, as assigned in the PDB [5], A–Z and a and b; these help further distinguish them, and they are listed in Table A1 for clarity [5].

The arrangement and labeling of the subunits in $A_{1}$ and $B_{1}$ of the proteasome is counterclockwise with respect to the arrangement in $A_{2}$ and $B_{2}$ (Figure 6). In chains D and R in the $B_{1}$ and $B_{2}$ rings, respectively, internal residues were missing (residues 118–124) so these were constructed in silico using CHARMM [14] and geometry optimized using 50 steepest descent (SD), followed by 100 adopted basis Newton-Raphson (ABNR) energy minimization steps. Hydrogen atoms were added using H-build from CHARMM [14]. The N-termini of each subunit was capped with a NH${}_{3}^{+}$ group and the C-termini were capped with a COO${}^{-}$. In addition, eight magnesium ions, two chlorine atoms, and 1520 water molecules, as found in the crystal structure, were included in the model, yielding a total of 103,063 atoms. All water molecules were modeled using TIP3 water parameters [15].

As a smaller model system, only the $B_{1}$ ring was considered together with the syringolin inhibitor, SylA. Two starting structures were prepared: (1) the inhibitor located at the binding site in subunit $\beta_{1}$ (corresponding to chain N) and (2) the inhibitor located in the center of the $B_{1}$ ring. Structure 1 was constructed as follows. The lower-resolution PDB structure containing the co-crystallized inhibitor (2ZCY [6]) was superimposed on the higher-resolution apo 5CZ4 structure [5] and the coordinates of the atoms belonging to SylA located at the $\beta_{1}$ subunit (chain N) were saved. The model was then constructed by merging the coordinates of the 20S CP from 5CZ4 with the SylA coordinates from 2ZCY. After modeling, any close contact (2.5 Å) water interactions were removed (47 close-contact waters), resulting in a total of 103,131 atoms. Additional close contacts were initially relaxed using 50 steepest descent (SD), followed by 50 ABNR energy minimization steps resulting in a 0.59 Å RMSD from the crystal protein backbone atoms. To construct structure 2, the inhibitor was positioned in the center of the $B_{1}$ ring. This position is assumed to be a non-biased starting position. Since the ring opening is on the order of 13 Å, [10] the substrate could pass through the center of the cylinder annulus, such that it arrives in approximately the center of the proteolytic chamber of the $B_{1}$ ring. As modeled, the initial distance from the C${}_{4}$ of SylA (see Figure 3 for atom labels) to Thr1-O${}^{\gamma }$ in each of the three active sites in $\beta_{1}$, $\beta_{2}$, and $\beta_{5}$ is approximately 27 Å, 34 Å, and 40 Å, respectively. For both models 1 and 2, any water molecule from the 5CZ4 PDB structure that was overlapping with the SylA molecule was deleted. In the case of structure 1, with SylA near the active site, modeling resulted in the deletion of 49 overlapping waters. In the case of structure 2, with SylA in the center of the ring, very few crystal structure water molecules are contained in this region so only seven overlapping water molecules were removed.

### 3.2. Potential Energy Function and Energy Minimization

**Figure A1:**
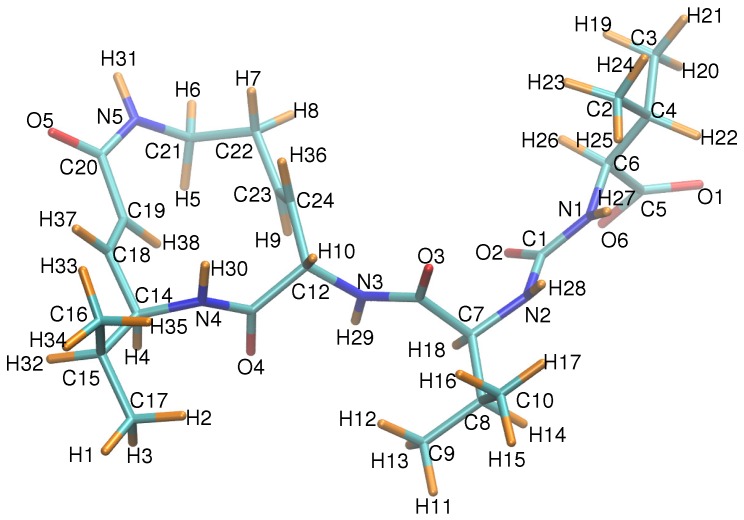
SylA is depicted with each atom labeled according to the numbering scheme of Table A2. Carbons are shown in cyan, oxygens in red, nitrogens in blue, and hydrogens in orange. Note that atom numbering here was only relevant for the charge parametrization procedure and differs from that of, e.g., Ref. [6]; C${}_{18}$ corresponds to C${}_{4}$.

The protein system was treated using the CHARMM36 parameter set for the protein [16,17] and the TIP3P model for water molecules [15]. For the syringolin inhibitor SylA, initial CHARMM parameters for the SylA were generated with the CHARMM General Force Field (CGenFF) (version 1.0.0) [18]. Next, the charges of the heteroatoms atoms (N and O) were optimized in the following manner. At each of the twelve hetero sites (Figure 3), an individual water molecule was constructed such that a colinear hydrogen bond was formed between the water molecule’s oxygen and the heteroatom. The resulting hydrogen bond energy and bond length were calculated [HF/6-31g* with a gradient tolerance of 0.00000001 kcal/mol/Å] with the GAMESS suite in CHARMM [19,20]. The resulting set of charges was then used to recalculate the charges for the entire molecule. The final set of SylA atomic charges, corresponding to atoms as shown in Figure A1, is listed in Table A2. Energy minimization was performed using the ABNR routine in CHARMM [14].

### 3.3. Molecular Dynamics

54 ns relaxation molecular dynamics were carried out for the 20S CP. The cylindrical structure as modeled with water molecules from the crystal structure, was aligned with the ring centers along the *Z*-axis and placed in a rectangular box (175 × 175 × 202 Å${}^{3}$) containing explicit water molecules (481902 total number of atoms, 126385 TIP3 water molecules [15]).

MD simulations were performed with the SylA inhibitor present using the $B_{1}$ ring as a model system for the proteasome. The ring, as modeled with inhibitor and water molecules from the crystal structure, was aligned with the *Z*-axis in the ring center normal to the ring plane and placed in a rectangular box (150 × 150 × 100 Å${}^{3}$) containing approximately 71,300 explicit TIP3 water molecules [15]. For structure 2, in which the inhibitor was positioned in the center of the $B_{1}$ ring, three independent simulations were carried out, each 10 ns in length.

To simulate a continuous system, periodic boundary conditions were applied. Electrostatic interactions were summed with the Particle Mesh Ewald method [21] (∼1.5 Å grid point spacing). The MD simulations used an integration time step of 2 fs and a non-bonded cutoff of 16.0 Å. The temperature (310 K) was controlled using Langevin dynamics, with a collision frequency of 20 ps${}^{-1}$ and isotropic position scaling to maintain pressure (1 atm). The SHAKE algorithm was used to constrain all bonds to hydrogen atoms [22]. Heuristic testing was performed at each time step to evaluate whether the non-bonded pair list should be updated.

### 3.4. Electrostatic Energy Calculations

The electrostatic energy of each of the 28 protease ring subunits was determined numerically by solving the linearized Poisson-Boltzmann equation (LPBE) using the Adapted Poisson-Boltzmann Solver (APBS) [23]. The calculation of electrostatic energies depends first and foremost on the quality of the molecular structure that is being treated [24]. For a particular titration state, the atomic charges and radii are assigned according to the selected force field. Here, the CHARMM22 force field was used, including grid-based energy correction map (named CMAP) terms for protein backbone Φ,Ψ dihedral angles and side-chain torsion potentials [17]. Assuming the structure of the biological system is reliable, solving the LPBE relies on the discretization of the PB equation and determining the electrostatic potential on a grid. The grid spacing, which can affect the solution of the LPBE, is chosen to maximize the resolution across the system of interest. Here the proteolytic ring has approximate dimensions of 110 Å × 110 Å × 50 Å. The number of grid points in the {*x*,*y*,*z*} dimensions for each calculation was 417, 449, and 225, respectively, resulting in grid spacings of 0.26 Å, 0.24 Å, and 0.22 Å, in the {*x*,*y*,*z*} directions, respectively. In comparison, the 30S small ribosomal subunit (containing 88,000 atoms) filling a box of dimension 200 Å${}^{3}$ was treated using the APBS method with a resolution of 0.41 Å [23]. APBS relies on a 10${}^{-6}$ error tolerance in the calculated potential. This tolerance has been observed to give good accuracy in APBS calculated energies; nonetheless, a comparison of electrostatic energies calculated using the same methodology typically results in the most meaningful values [23].

Each of the four rings was analyzed separately. Each ring was treated with the dielectric constant of the solvent set to ϵ=80 and inside the protease volume to ϵ=4. The choice of ϵ=4 for inside the protein volume has demonstrated reasonably good agreement with experiments while also accounting for polarization and small backbone fluctuations [23,25,26]. The electrostatic potential experienced by each ring subunit was calculated as follows: for the arrangement of six of the seven ring units, the potential was tabulated by summing over all charges while the charges of the seventh subunit were set to 0. The electrostatic potential energy of the seventh subunit was calculated as a product of its charges with the electric field generated by the remaining six ring subunits. This procedure was repeated seven times within one ring. In total, 28 calculations were carried out, one for each CP ring subunit.

### 3.5. Solvation Binding Energy Calculations

**Figure 9:**
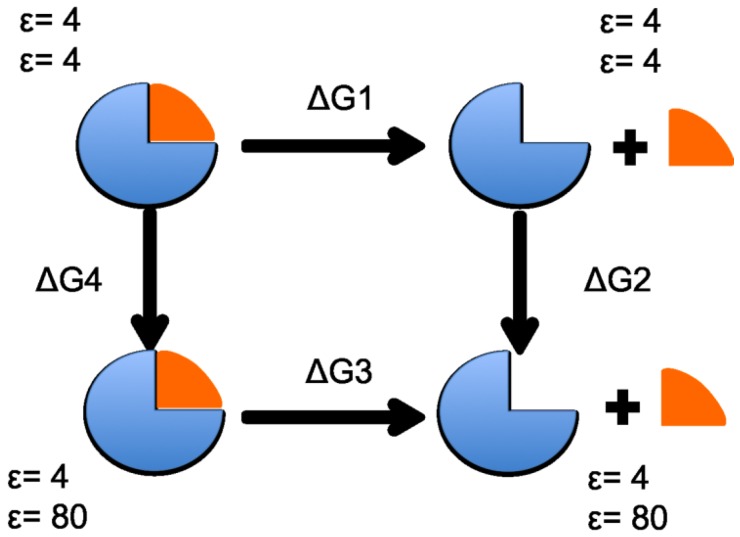
The thermodynamic cycle depicts the steps involved in the calculation of binding free energy of a protein subunit (orange) in the environment of a protein complex (blue).

To estimate the solvation binding energy of each ring subunit, the components of the standard thermodynamic energy cycle were calculated for each subunit according to the scheme in Figure 9. The term ΔG1 is the free energy difference, in an environment with a homogeneous dielectric constant (ϵ=4), between the product (ring and with subunit separated) and the reactant (complete ring). ΔG2 and ΔG4 refer to the free energy difference gained from moving the solute from an environment with a homogeneous dielectric constant (ϵ=4, ϵ=4) to a heterogeneous dielectric environment (ϵ=4 for the solute, ϵ=80 for the solvent). The binding energy, −ΔG3, is then calculated from ΔG3=ΔG1+ΔG2−ΔG4.

As for the electrostatic energy calculations, each of the four heptameric rings was analyzed separately. Therefore, the thermodynamic cycle in Figure 9 was calculated for each of the seven subunits in each heptameric ring, resulting in a total of 28 sets of calculations. ΔG4 and ΔG2 were determined by solving the linearized Poisson-Boltzmann equation using the Adapted Poisson-Boltzmann Solver (APBS) [23]. Each ring (aligned with the ring center along the *Z*-axis) was treated with the same number of grid points: 417, 449, and 225 for the {*x*,*y*,*z*} directions, respectively. ΔG1 was calculated using CHARMM [14].

## 4. Conclusions

As the delivery of substrates to the active sites in the 20S CP is ATP-independent, the process must be energetically favorable. The forces guiding substrates from the CP’s outer A rings to the inner proteolytic chambers arise from the molecular architecture of the subunit arrangement and from the interaction of the substrate with the CP subunits. Here, we present the results of 54 ns MD simulations of the 20S CP and of the smaller model system, the proteolytic $B_{1}$ ring, with the SylA inhibitor. Over the sampled time, the CP demonstrates dynamical contraction behavior, evidenced by RMS deviations, and analysis of A and B ring electrostatics and solvation binding energies indicate variations within the four heptameric rings that may be one source of these conformational changes. Nonetheless, extending the simulation time is necessary to determine whether these changes are due to relaxation or to a “breathing” motion.

Electrostatic energy calculations have located specific regions within the individual rings, for example between $\beta_{1}$ and $\beta_{2}$ of the $B_{1}$ ring, that are characterized by a high electrostatic energy gradient. Further analyses of the electrostatic and binding energies of the individual ring subunits reveal variations within the subunits of each ring. These variations may indicate dynamical mobility of the proteasome CP that accommodates substrates in regions containing the catalytically active centers. Here, we have not considered the possibility of non-standard protonation states of titratable residues. As protonation patterns may affect calculated electrostatic properties, future studies should include a comprehensive review of assigned protonation states.

To carry out simulations with SylA, we have computed and parametrized SylA charges. MD simulations of the SylA inhibitor with the smaller proteasome model, the $B_{1}$ ring, provide insight into the source of dynamical behavior of the inhibitor. In the first simulation, at t = 0 ns, SylA is located at the active site of $\beta_{1}$, and it remains at its initial position throughout the 10 ns trajectory. In simulations 2–4, in which SylA at t = 0 ns was simulated in the center of the ring, an overall stabilization due to electrostatic and vdW energies is observed as the inhibitor migrates toward the proteasome wall. This migration occurs on a time scale of approximately 4 ns. Close proximity to protein chains, regardless of the presence of an active site, is responsible for stabilizing the inhibitor position through favorable electrostatic and van der Waals energies between SylA and the ring subunits.

In the current study, we considered two scenarios for the $B_{1}$ ring, namely (1) at t = 0 ns, the SylA inhibitor is located at the $\beta_{1}$ active site and (2) at t = 0 ns, SylA is located in the center of the ring. In future simulations, additional studies should examine the interaction of SylA with the other two catalytic sites, i.e., at t = 0 ns SylA is located directly at either the $\beta_{2}$ or $\beta_{5}$ active site. In addition, the scenario in which all three active sites are occupied with SylA should be examined. The presence of more than one inhibitor in the proteolytic chamber likely increases the probability that the inhibitors spend time near an active site.

The “dissection” approach–studying a single ring of the CP–has been used here in the investigation of inhibitor dynamics. In the future, larger model systems, for example in which both catalytic rings $B_{1}$ and $B_{2}$ are simulated together, may provide us with more insight into the behavior of inhibitor dynamics within the rings. Extending the duration of MD simulations would also provide us with further insight into large-scale conformational changes in the CP structure, as well as substrate behavior inside the proteolytic chamber. Here, using nanosecond simulations of a single proteolytic ring, we have analyzed the forces driving substrate dynamics, a necessary first step for engineering proteasome inhibitors.

## Abbreviations

The following abbreviations are used in this manuscript:

NPC	Niemann-Pick type C
NPC1	NPC protein 1
NPC2	NPC protein 2
MD	Molecular dynamics
TMD	Targeted molecular dynamics
NTD	N-terminal domain
MLD	Middle luminal domain
CTD	C-terminal domain

## Appendix A

**Table A1:** Electrostatic potential energy, binding energy (each given in [kcal/mol]), and average B-factor of each proteasome ring subunit. The corresponding chain symbol (A–Z, a, and b) from the PDB file 5CZ4.pdb is listed in the parentheses after each subunit. For each of the two B rings, the three subunits ($\beta_{1}$, $\beta_{2}$, $\beta_{5}$) containing the active sites are marked in bold. The table arrangement of rings is according to the spatial arrangement in the proteasome: $A_{1}$, $B_{1}$, $B_{2}$, $A_{2}$.

Ring	Subunit (pdb Label)	Electrostatic Energy	Binding Energy	Average B-Factor
$A_{1}$				
	$\alpha_{1}$ (G)	−136	−141	52
	$\alpha_{2}$ (A)	−120	-203	52
	$\alpha_{3}$ (B)	−196	−177	50
	$\alpha_{4}$ (C)	−173	−160	58
	$\alpha_{5}$ (D)	−151	−3	60
	$\alpha_{6}$ (E)	−169	−68	66
	$\alpha_{7}$ (F)	−158	−49	61
		Tot: −1102	Tot: −801	Ave: 57
$B_{1}$				
	$\beta_{1}$ (N)	−97	−174	46
	$\beta_{2}$ (H)	−235	−88	52
	$\beta_{3}$ (I)	−234	−118	45
	$\beta_{4}$ (J)	−99	−163	49
	$\beta_{5}$ (K)	−111	−128	47
	$\beta_{6}$ (L)	−147	−135	49
	$\beta_{7}$ (M)	−152	−113	49
		Tot: −1075	Tot: −919	Ave: 48
	$\beta_{1}^{\prime }$ (V)	−220	−56	56
	$\beta_{2}^{\prime }$ (b)	−93	−153	47
	$\beta_{3}^{\prime }$ (a)	−147	−84	48
	$\beta_{4}^{\prime }$ (Z)	−144	−121	49
	$\beta_{5}^{\prime }$ (Y)	−106	−120	38
	$\beta_{6}^{\prime }$ (X)	−96	−158	35
	$\beta_{7}^{\prime }$ (W)	−222	−123	48
		Tot: −1028	Tot: −815	Ave: 46
$A_{2}$				
	$\alpha_{1}^{\prime }$ (U)	−127	−112	57
	$\alpha_{2}^{\prime }$ (T)	−146	−41	71
	$\alpha_{3}^{\prime }$ (S)	−151	−77	76
	$\alpha_{4}^{\prime }$ (R)	−151	−18	64
	$\alpha_{5}^{\prime }$ (Q)	−163	−169	78
	$\alpha_{6}^{\prime }$ (P)	−177	−188	59
	$\alpha_{7}^{\prime }$ (O)	−111	−205	62
		Tot: −1026	Tot: −810	Ave: 66

**Table A2:** SylA charge parameters are listed with heavy atoms in bold; groups are separated.

Atom	Type	Charge
**C1**	CG2O6	0.202
**N1**	NG2S1	−0.512
H27	HGP1	0.308
**O2**	OG2D1	−0.273
**C2**	CG331	−0.271
H23	HGA3	0.090
H24	HGA3	0.090
H25	HGA3	0.090
**C3**	CG331	−0.271
H19	HGA3	0.090
H20	HGA3	0.090
H21	HGA3	0.090
**C4**	CG311	−0.092
H22	HGA1	0.090
**N2**	NG2S1	−0.828
H28	HGP1	0.490
**C7**	CG311	0.051
H18	HGA1	0.090
**C8**	CG311	−0.090
H14	HGA1	0.090
**C9**	CG331	−0.271
H11	HGA3	0.090
H12	HGA3	0.090
H13	HGA3	0.090
**C10**	CG331	−0.271
H15	HGA3	0.090
H16	HGA3	0.090
H17	HGA3	0.090
**C5**	CG2O3	0.620
**C6**	CG311	0.048
**O1**	OG2D2	−0.560
**O6**	OG2D2	−0.560
H26	HGA1	0.099
**C11**	CG2O1	0.129
**O3**	OG2D1	−0.140
**N3**	NG2S1	−0.448
H29	HGP1	0.332
**C11**	CG2O1	0.129
**O3**	OG2D1	−0.140
**N3**	NG2S1	−0.448
H29	HGP1	0.332
**C13**	CG2O1	0.305
**O4**	OG2D1	−0.309
**N4**	NG2S1	−0.339
H30	HGP1	0.233
**C12**	CG311	0.044
H10	HGA1	0.090
**C14**	CG311	0.056
H4	HGA1	0.090
**C15**	CG311	−0.078
H32	HGA1	0.090
**C18**	CG2DC1	−0.156
H37	HGA4	0.150
**C19**	CG2DC1	−0.161
H38	HGA4	0.150
**C16**	CG331	−0.273
H33	HGA3	0.090
H34	HGA3	0.090
H35	HGA3	0.090
**C17**	CG331	−0.273
H1	HGA3	0.090
H2	HGA3	0.090
H3	HGA3	0.090
**C20**	CG2O1	0.443
**O5**	OG2D1	−0.392
**N5**	NG2S1	−0.522
H31	HGP1	0.312
**C21**	CG321	−0.011
H5	HGA2	0.090
H6	HGA2	0.090
**C22**	CG321	−0.184
H7	HGA2	0.090
H8	HGA2	0.090
**C23**	CG2D1	−0.146
H9	HGA4	0.150
**C24**	CG2D1	−0.163
H36	HGA4	0.150
